# Imbalance of bladder neurohomeostasis by Myosin 5a aggravates diabetic cystopathy

**DOI:** 10.1186/s10020-025-01140-6

**Published:** 2025-03-10

**Authors:** Yao Zhang, Jiao Zhang, Jiaye Liu, Lang Liang, Na Zhou, Shaochan Liang, Jingyi Huang, Ming Hong, Rui Wang, Siyuan Xu, Chiming Gu, Bo Tan, Hongying Cao

**Affiliations:** 1 School of Pharmaceutical Sciences, Guangzhou University of Chinese Medicine, No. 232, Waihuan East Road, Guangzhou Higher Education Mega Center, Panyu District, Guangzhou, 510006 Guangdong China; 2 Research Centre of Basic Integrative Medicine, School of Basic Medical Sciences, Guangzhou University of Chinese Medicine, No. 232, Waihuan East Road, Guangzhou Higher Education Mega Center, Panyu District, Guangzhou, 510006 Guangdong China; 3 The Second Affiliated Hospital of Guangzhou University of Chinese Medicine (Guangdong Provincial Hospital of Traditional Chinese Medicine), Guangzhou, 510006 Guangdong China

**Keywords:** Myosin 5a, Neurotransmission, Overactive bladder, Diabetic cystopathy, DBA mice

## Abstract

**Background:**

Diabetic cystopathy (DCP) is linked to bladder nerve conduction disorders, with diabetes-induced neuropathy impairing nerve signal transmission and causing bladder dysfunction. Myosin 5a, vital for neuronal transport, has been linked to neurological disorders, though its role in DCP remains unclear. The objective of this study was to investigate whether Myosin 5a plays a potential regulatory role in Diabetic Cystopathy.

**Methods:**

Bladder strips from diabetic rats were use to assess heightened responsiveness to external stimuli. Urodynamic assessments were conducted to track the progression of bladder voiding dysfunction over time, following streptozotocin (STZ) injection. Single-cell RNA-Seq mining was employed to identify associations between Myosin 5a and bladder overactivity. Cellular and tissue analyses were performed to determine the co-localization of Myosin 5a with neurotransmitter-related proteins. The impact of Myosin 5a knockdown on ChAT and SP expression in bladder neurons was also evaluated. Additionally, Myosin 5a-deficient DBA mice were studied for voiding function and sensitivity to stimuli. Student's t-test (two-tailed) or Mann–Whitney’s U test analysis of variance was used to analyze the difference between groups.

**Results:**

Bladder strips from diabetic rats exhibit increased responsiveness to external stimuli, with urodynamic assessments showing a progressive decline in bladder function, culminating in overactivity by the fourth week post-STZ injection. Co-localization of Myosin 5a with neurotransmitter-related proteins was observed, and the knockdown of Myosin 5a in bladder neurons led to a significant reduction in ChAT and SP expression. Myosin 5a-deficient DBA mice exhibited abnormal voiding function and reduced sensitivity to stimuli, along with significant downregulation of SLC17A9. Single-cell RNA-Seq analysis revealed a significant link between Myosin 5a and bladder overactivity, with Myosin 5a expression escalating in tandem with the severity of bladder dysfunction.

**Conclusions:**

Myosin 5a's dysregulation in diabetic rats may worsen bladder overactivity, suggesting its potential as a therapeutic target for diabetic OAB.

**Supplementary Information:**

The online version contains supplementary material available at 10.1186/s10020-025-01140-6.

## Introduction

Diabetes mellitus (DM) is a chronic metabolic condition that poses a significant global health challenge, reaching epidemic proportions with an estimated overall prevalence of up to 11.6% in China (Alam et al. 2014). As a key component of metabolic syndrome, DM significantly affects multiple organ systems, including the kidneys and bladder (Grundy et al. 2005; Akhtar et al. 2020). The pathogenesis of diabetic cystopathy (DCP) is complex and involves various factors, including alterations in detrusor muscle physiology, neuronal impairment, and urothelial dysfunctions (Goepel et al. 2002). Diabetic cystopathy, also referred to as diabetic bladder dysfunction (DCP), is a prevalent urological complication encompassing overactive bladder, voiding dysfunction, and urinary retention. The prevalence of diabetic cystopathy varies widely, with estimates ranging from 25 to 90%. Epidemiological research indicates that overactive bladder (OAB) is significantly more common in individuals with DM compared to the general population. The persistent and urgent need to urinate can result in feelings of frustration and helplessness, ultimately leading to a diminished quality of life (Type II 2016; Golbidi and Laher 2010).

The neural control of the bladder relies on two primary components: parasympathetic nerves from the sacral spinal cord, which stimulate detrusor muscle contraction, and sympathetic nerves from the thoracolumbar spinal cord, which inhibit detrusor muscle activity (Ochodnicky et al. 2013). Diabetes mellitus (DM) induces pathological changes in the peripheral nerves that innervate the bladder. These changes include motor neuron atrophy, gliosis with vacuolar degeneration, cytoplasmic vacuolation, and nuclear necrosis, often presenting as bead-like or spindle-shaped fragmentation (Agochukwu-Mmonu et al. 2020). Damage to both afferent and efferent bladder fibers leads to impaired bladder sensation and reduced bladder contraction (Katona and Weis 2017). Furthermore, research has shown that decreased levels of nerve growth factor (NGF) in the bladder and lumbosacral dorsal root ganglia are associated with impaired bladder sensation and dysfunction in Aδ and C fiber afferent pathways (Steers and Tuttle 2006).

The development of bladder contraction dysfunction may be associated with cholinergic nerve damage, reduced acetylcholinesterase activity, and dysregulation of non-cholinergic, non-adrenergic (NANC) neurotransmitter homeostasis. Acetylcholine at the neuromuscular junction promotes bladder emptying by inducing smooth muscle contraction. Additionally, cholinergic nerves are crucial for sensing bladder fullness, transmitting this information to the central nervous system to regulate the urge to void and overall bladder function (Chaudhury et al. 2011; Moutin et al. 2012; Bodor et al. 2014). NANC pathways are critically involved in the pathogenesis of various disorders, contributing to approximately 40% of neural conduction in the diabetic bladder (Röder et al. 2008, 2010). Peptidergic nerves, including those releasing substance P (SP), are crucial for bladder function. SP modulates the sensory pathways of bladder distension through neurokinin receptors, which are essential for sensory and reflexive bladder control. Vesicular nucleotide transporter, which is encoded by the SLC17A9 gene is critical for purinergic nerve conduction, as it transports ATP and other nucleotides into neuronal vesicles. This stored ATP is released into the synaptic cleft, activating purinergic receptors that regulate neurotransmission, pain signaling, and neuroinflammation. SLC17A9 disruptions can impair ATP release and neural communication in diabetic bladder dysfunction. However, the molecular mechanisms underlying the imbalance of neural homeostasis in DCP remain unclear.

Myosin motors represent a large superfamily of motor proteins characterized by their distinct molecular structures and cargo specificities. Several unconventional Myosins, such as Myosins 2, 5a, 5b, and 6, have been implicated in the processes of vesicular exocytosis and endocytosis, particularly within neuronal cells (Bridgman 2009). Myosin 5a, in particular, is extensively expressed in both presynaptic and postsynaptic neurons throughout the central and peripheral nervous systems (Takagishi et al. 2005a). It facilitates the movement of neurotransmitter-containing vesicles and organelles along actin filaments, which is essential for synaptic function and neuronal integrity. Additionally, it ensures the proper localization of organelles such as mitochondria, thereby contributing to cellular energy balance and stress responses (Hartman et al. 2011). Myosin 5a also plays a role in axon guidance and growth, underscoring its significance in neural development and stability. Dysfunctional Myosin 5a disrupts the stability of nicotinic ACh receptors, leading to impaired neuromuscular junction function (Saneyoshi et al. 2010). It also plays a role in the synaptic vesicle transport of cholinergic neurons (Yoshii et al. 2013). Besides, Myosin 5a associates with SLC17A9-stained vesicles and possibly transports them to the varicosity membrane for exocytosis, and purinergic inhibitory neurotransmission is impaired in Myosin 5a-deficient mice. However, the effect of Myosin 5a on bladder nerve homeostasis in DCP rats is still unclear.

In this study, we established a stable diabetic rat model through injection of streptozotocin (STZ). Observations revealed that, alongside elevated blood glucose levels, these rats developed abnormal micturition patterns and increased detrusor sensitivity. Even by the fourth week, the model animals exhibited bladder overactivity. To elucidate the underlying mechanisms, we performed a comprehensive analysis of single-cell RNA sequencing data from patients with overactive bladder (OAB). This analysis revealed a close association between the pathogenesis of OAB and Myosin as well as neurotransmission pathways. Further investigation demonstrated the co-localization of Myosin 5a with neurotransmitter-related proteins. Utilizing siRNA-mediated knockdown techniques and Myosin 5a-deficient DBA mice, we validated that aberrant expression of Myosin 5a in the context of diabetes significantly disrupts bladder nerve homeostasis, thereby exacerbating diabetic cystopathy. Our findings enhance the understanding of the pathophysiological mechanisms underlying diabetic bladder disease and uncover a previously unrecognized role of Myosin 5a in modulating bladder nerve homeostasis, offering new theoretical insights for the clinical management of DCP.

## Methods

### Ethics approval and consent to participate

The protocols involved in this study were following the rules and guidelines of the Experimental Animal Centre of Guangzhou University of Chinese Medicine and were approved by the Guangzhou University of Chinese Medicine Animal Care and Use Ethics Committee (Ethics approval number: ZYD-2021-180).

### Animal model

Male Sprague–Dawley rats, aged 6 weeks and weighing 180–220 g, were randomly assigned to two groups using a random number table: DM and control groups. All rats were housed in a room with a temperature of 25 ± 2℃ and a 12-h light–dark cycle. The DM rats received an injection of STZ (65 mg/kg). Fasting blood glucose (FBG) levels were measured 72 h after the STZ injection, and rats with FBG ≥ 8.3 mmol/L were considered the DM model rats. The DM rats were then randomly divided into three groups based on the duration of the model: 2‐, 3‐, and 4‐week model groups. Similarly, the control rats were also divided into corresponding groups.

As controls, male wild-type (WT) C57BL/6 J mice aged 4–6 weeks with solid black coat color were used. Male DBA/2 J mice served as the models of *Myosin 5a* deficiency. DBA/2 J mice, which have similar mutations in Myo5a and no apparent phenotypic differences compared to DBA/1 J mice, were chosen based on preliminary data. The DBA mice (dba) were originally raised at National Institutes of Health and Jackson Laboratories through intercrossing homozygous for the coat colors dilute (d/Myo5a), brown (b/Tyrp1), non-agouti (a), making them one of the most ancient of all inbred strains.

### Measurements of weight and FBG test

The weight and fasting blood sugar of the rats were measured at 2, 3, and 4 weeks. Before the tests, the rats underwent a 12-h fasting period. Using the ACCU-CHEK Active blood glucose meter, a test strip was inserted. The rat's tail tip was pricked with a tiny needle from the meter, and a drop of blood was obtained and applied to the test strip's edge. The blood sugar reading was then swiftly recorded, yielding valuable data for the study.

### Cystometry

General anesthesia was induced by intraperitoneal injection of ethyl carbamate (1 g/kg). The bladder of the animal was surgically exposed, and a 25-gauge needle was inserted into the dome of the bladder. This needle was connected to a fluid-filled pressure line on one end and an injection pump (Harvard Apparatus) on the other via a three-way adapter. The pressure line was linked to a urodynamic measuring device (Laborite Delphis 94-R01-BT, Canada). Sterile saline was infused into the bladder at a constant rate (3 mL/h) based on our previous studies (Zhang et al. 2022). Once urine appeared in the urethral orifice of the rats, the micro syringe pump was turned off, and the bladder pressure line automatically recorded the data with a computer.

The following parameters were measured: micturition volume (MV), maximum voiding pressure (MVP), residual volume (RV), maximum bladder capacity (MBC), bladder compliance (BC), and voided efficiency (VE). RV was manually drained and measured using a 1 ml syringe. MBC was calculated as the infused volume immediately before detrusor contraction. BC was determined as (MBC/MVP) × 100%, while VE was calculated as (MBC − RV) / MBC × 100%. To minimize discrepancies, the mean values of three voiding cycles of each rat were utilized. The assay was conducted three times for each rat.

### *Assessment of bladder smooth muscle contractility *in vitro

Bladders harvested at 2, 3, and 4 weeks from both control and DM groups were placed in oxygenated Krebs solution. Each bladder was then longitudinally cut into four urothelium-intact strips, each measuring 5–7 mm × 2 mm. These tissue strips were suspended in a 5 mL organ bath containing oxygenated Krebs solution, maintained at 37 °C, and continuously aerated with 95% oxygen and 5% carbon dioxide. The strips were equilibrated for 60 min at a resting tension of 1.0 g.

Subsequently, the contractile response to various stimuli was measured. This included electrical-field stimulation (EFS) at different frequencies (2, 4, 8, 16, 32, and 64 Hz; 40 V; and 0.5 m/s pulse duration for 10 s), carbachol (ranging from 10^–8^ to 10^–5^ M), and KCl (120 mM). The carbachol curves were cumulative, with the base value used in the analysis being the baseline immediately before the beginning of the curve. After the experiments, the weight and length of each DSM strip were recorded.

### Histology

The rats were euthanized, and their bladders were harvested and weighed at 2, 3, and 4 weeks. The bladders were then fixed in 4% paraformaldehyde solution and embedded in paraffin. Transverse sections of the bladders (6 μm thick) were obtained and stained using hematoxylin and eosin (H&E) as well as Masson's trichrome (100 × magnification). Bladder wall thickness (BWT) was determined from the H&E images, while the smooth muscle-to-collagen ratio was calculated from the Masson's trichrome images. All images were processed and analyzed using ImageJ software. To ensure unbiased evaluation, the histology slides were examined without knowledge of the group allocation.

### Real-time qPCR

Total ribonucleic acid (RNA) was extracted from the bladder using TRIzol reagent (Thermo Fisher Scientific). The purity and concentration of RNA samples were evaluated through ultraviolet absorption at 260 and 280 nm, with A260/A280 used as an indicator of purity. For cDNA synthesis, Fastking gDNA Dispelling RT SuperMix (TIANGEN, China) was employed with the total RNA. Subsequently, a qPCR assay was conducted on an ABI Prism 7500 system (Applied Biosystems, USA) using Talent qPCR PreMix (SYBR Green) (TIANGEN, China). Synthetic oligonucleotide primers, listed in Table 1, were designed for amplifying cDNA corresponding to the genes encoding Myosin 5a and GAPDH. The results are presented as mRNA levels for each gene, normalized to *Gapdh* expression.

**Table 1:** Primers used in the present study

Gene	Primer (5′-3′)
*Myosin 5a*-F	ACGAACAGGCTCTTAGAATCCC
*Myosin 5a*-R	TGCTGTTGGCCGGTTGTTTTC
*Gapdh*-F	TGAGCATCTCCCTCACAATTCC
*Gapdh*-R	TTTTTGAGGGTGCAGCGAAC
*M3*-F	CCCACAGGCAGTTCTCGAA
*M3*-R	CCTCCTAGATGACCGTTTCGT
*P2* × *1*-F	ATTCGCTTTGATATCCTTGTGG
*P2* × *1*-R	GCCGATGGTAGTCATAGTAGGG
*Slc17a9*-F	GCTTCCTCAAGGCTATGATCTT
*Slc17a9*-R	AGGTCCTGAATGTTGACTGAAA

### Single-cell RNA-seq analysis

Gene expression profiles from human bladder tissues were acquired through the Gene Expression Omnibus (GEO) repository under accession numbers GSM4850577, GSM5733428, and GSE175526 (dataset GSM5340862-GSM5340866)). For normal control group, bladder tissue were obtained from a consented adult male donor who succumbed to traumatic brain injury. The procurement protocol was approved by the Ethics Committee for Clinical Research and Animal Trials of the First Affiliated Hospital of Sun Yat-sen University (Approval No. [2018]255) and conducted in accordance with the Declaration of Helsinki. For the disease group, the two datasets were integrated to constitute the disease samples and were approved by the Ethics Committee of Luohu District People’s Hospital (approval number not specified) and the Institutional Review Board of Beijing Hospital (Approval No. 2020BJYYEC-141-01), respectively.

All data processing procedures were conducted by R (version 4.4.0), the Seurat platform (version 5.1.0), and the Single Cell Pipeline platform (Hao et al. 2024; Wu et al. 2021; H Z 2023). Subsequently, the data underwent sequential quality control, integration, normalization, scaling, batch effect removal, cluster annotation, and based on the annotation results, differentially expressed genes identification, enrichment analysis, and gene set expression difference analysis were performed. The selection of all relevant parameters was based on previous studies (He et al. 2020; Luo et al. 2022; Su et al. 2022).

### Primary cultures of rat bladder neurons

An improved method based on a previously reported procedure (Wang et al. 2021) was used in this study. First, the materials were prepared, including Krebs solution, rinse media, neuron media A, and neuron media B. The bladder was obtained and washed with Krebs solution. Primary rat bladder cells were obtained through two digestion steps, and cold rinse media were used to stop the digestion process. The cells were then suspended in neuron media A and seeded onto 24-well plates with glass coverslips coated with poly-D-lysine and laminin feeder layer substrates. Cultures were maintained in a humidified chamber with 5% CO_2_ and 95% O_2_ at 37 °C. After 24 h, the media were replaced with neuron media B for serum-free culture, with subsequent media changes every 2 days.

### Knock-down

Small interfering RNA (siRNA) targeting the *Myosin 5a* gene was designed, synthesized, and added to the cultured cells after 72 h.

### Cell Immunofluorescence staining

After 5 days of growth, the medium was removed, and the cells were fixed with 4% PFA for 30 min. Subsequently, the cells were washed with PBS, and permeabilization was performed using PBS with 0.1% Triton X-100. Blocking was done with 10% goat serum, and primary antibodies against Myosin 5a, β-tubulin3, ChAT, SP, and SLC17A9 were added and incubated overnight. After washing, the appropriate secondary antibodies were added and incubated in the dark. Hoechst 33342 was used for nuclear staining. Finally, the cells were observed using a laser confocal microscope.

### Tissue immunofluorescence staining

Bladder tissue samples were prepared by removing the urothelium and fixing the harvested bladders in 4% PFA with 0.1 M PBS. Detrusor samples were obtained through microdissection and permeabilized using 0.3% Triton X-100 with 10% goat serum or BSA in PBS. The tissues were then incubated for 16 h at 4 °C with primary antibodies targeting Myosin 5a, ChAT, SP, and SLC17A9. After thorough washing, secondary antibodies conjugated with fluorescent tags (Alexa 488, Cy3, or Alexa 555) were applied for 2 h at room temperature. Laser scanning confocal microscopy was used to visualize and analyze the labeled proteins.

### Image analysis

A laser-scanning confocal microscope was used to capture the image. For cell observation, three cell slides were analyzed for each antibody, with two random zones per slide. Similarly, for tissue immunofluorescence staining, three detrusor sections were analyzed for each antibody, with two random zones per slide.

### Statistical analysis

Statistical analyses were conducted using IBM SPSS Statistics 26.0. Data are presented as mean ± SD. For experiments involving independent samples, a Student’s t-test (t-test) from the general linear model was used when the data met the assumptions of normality and homogeneity of variances (assessed by Shapiro–Wilk and Levene’s tests, respectively); otherwise, nonparametric analyses (Mann–Whitney U test) were employed. A significance level of *P* < 0.05 was used to determine statistical significance.

## Results

### Dynamic monitoring of blood glucose and body weight in diabetic rats

**Fig. 1:**
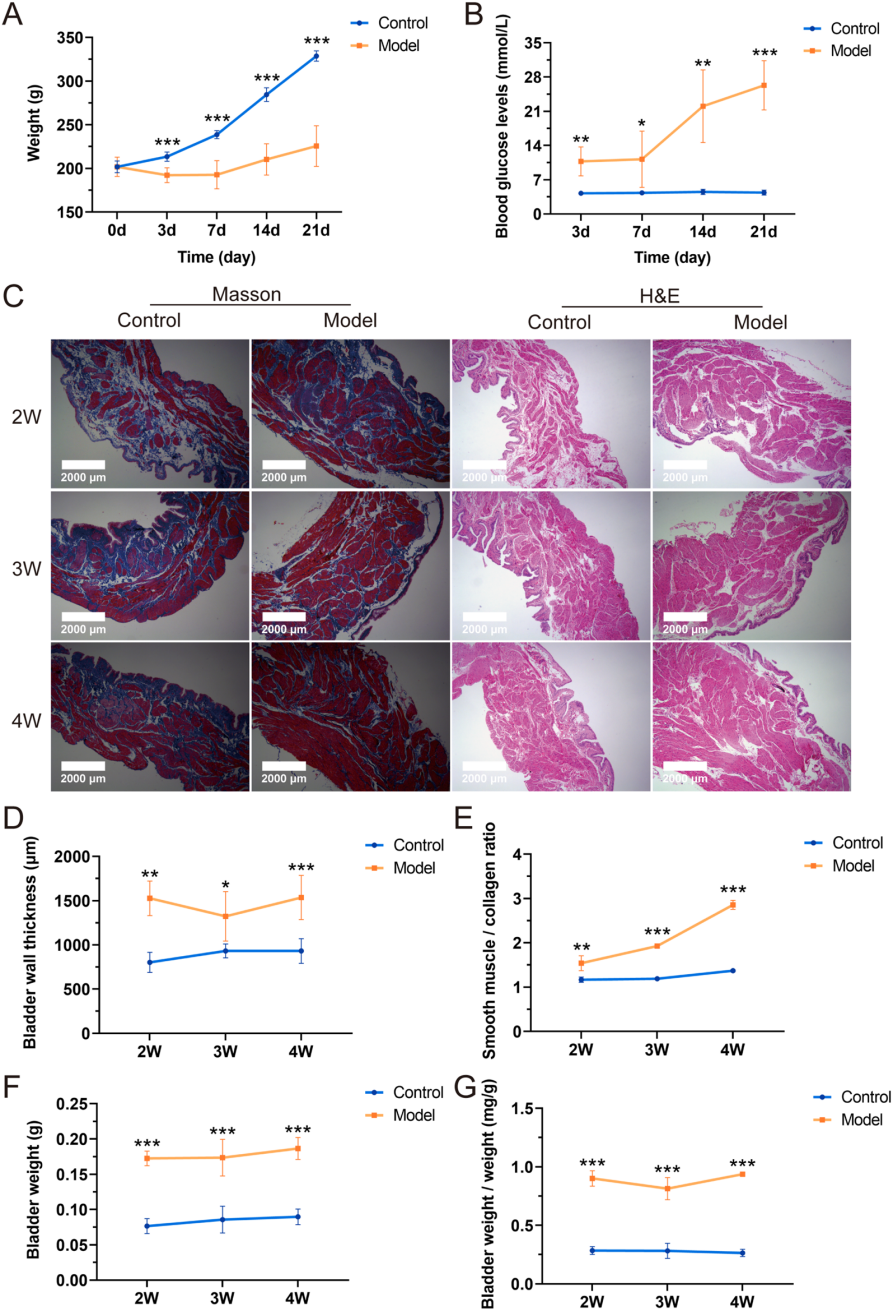
Digitalization images (100 ×) from Masson’s and H&E staining of DCP and control rats. **A** Weight (n = 6 rats/group, ***P = 0.000420, 0.000057, 0.000040, 0.000066 at 3d, 7d, 14d, and 21d respectively *vs.* control group); **B** Fasting glucose (n = 6 rats/group, ***P = 0.000066 at 21d, **P = 0.003823, 0.004922, 0.003885, 0.003885 at 3d, 14d, and *P = 0.0.032734 at 21d respectively *vs.* control group); **C** Digitalization images (100 ×) from Masson’s trichrome staining and H&E staining; **D** bladder wall thickness;** E** smooth muscle-to-collagen ratio; **F** bladder weight; **G** bladder-weight-to-body-weight ratio. Data are shown as the mean ± SD ($\overline{x }$±SD, n = 6 rats/group). Data were analyzed using t test or Mann–Whitney’s U test. For bladder wall thickness, **P = 0.003948 at 2W, *P = 0.017113 at 3W, ***P = 0.000422 at 4W respectively *vs.* the control group. For ratio of smooth muscle-to-collagen, **P = 0.001932 at 2W, ***P = 2.94E-13, 7.60E-12 at 3W and 4W respectively *vs.* control group. For bladder weight, ***P = 2.0171E-8, 0.000053, 2.1059E-7 *vs.* control group at 2W, 3W, 4W, respectively. For bladder-body weight ratio, ***P = 1.6943E-9, 4.8739E-7, 0.000006 *vs.* control group at 2W, 3W, 4W, respectively

In contrast to the control group, which demonstrated a consistent increase in body weight over a three-week period on a standard diet, the body weight of the model group rats remained relatively stable following STZ induction. By day three, the blood glucose levels in the model group rats had surpassed 8.3 mmol/L, aligning with the criteria for mild hyperglycemia (Furman 2021). Additionally, blood glucose concentrations in this group exhibited a progressive increase over time (*P* < 0.05) (Fig. 1A, B).

### Dynamic morphological assessment of the bladder in diabetic rats

Subsequently, the structural changes in the bladders of diabetic rats using H&E and Masson’s trichrome staining were analyzed (Fig. 1C). Morphometric analysis of bladder demonstrated a significant increase in bladder thickness in diabetic rats at 2, 3, and 4 weeks compared to control rats (*P* < 0.05) (Fig. 1D). Additionally, the ratio of smooth muscle to collagen in the bladder tissues of diabetic rats showed a progressive increase over 2, 3, and 4 weeks (*P* < 0.01) (Fig. 1E). Furthermore, bladder weight in diabetic rats was significantly higher than that in control rats at 2, 3, and 4 weeks (*P* < 0.001) (Fig. 1F). The bladder weight-to-body weight ratio was also significantly elevated in diabetic rats compared to controls at these time points (*P* < 0.001) (Fig. 1G).

### Enhanced response of isolated bladder muscle strips to EFS, CCh, and KCl stimulation in diabetic rats

**Fig. 2:**
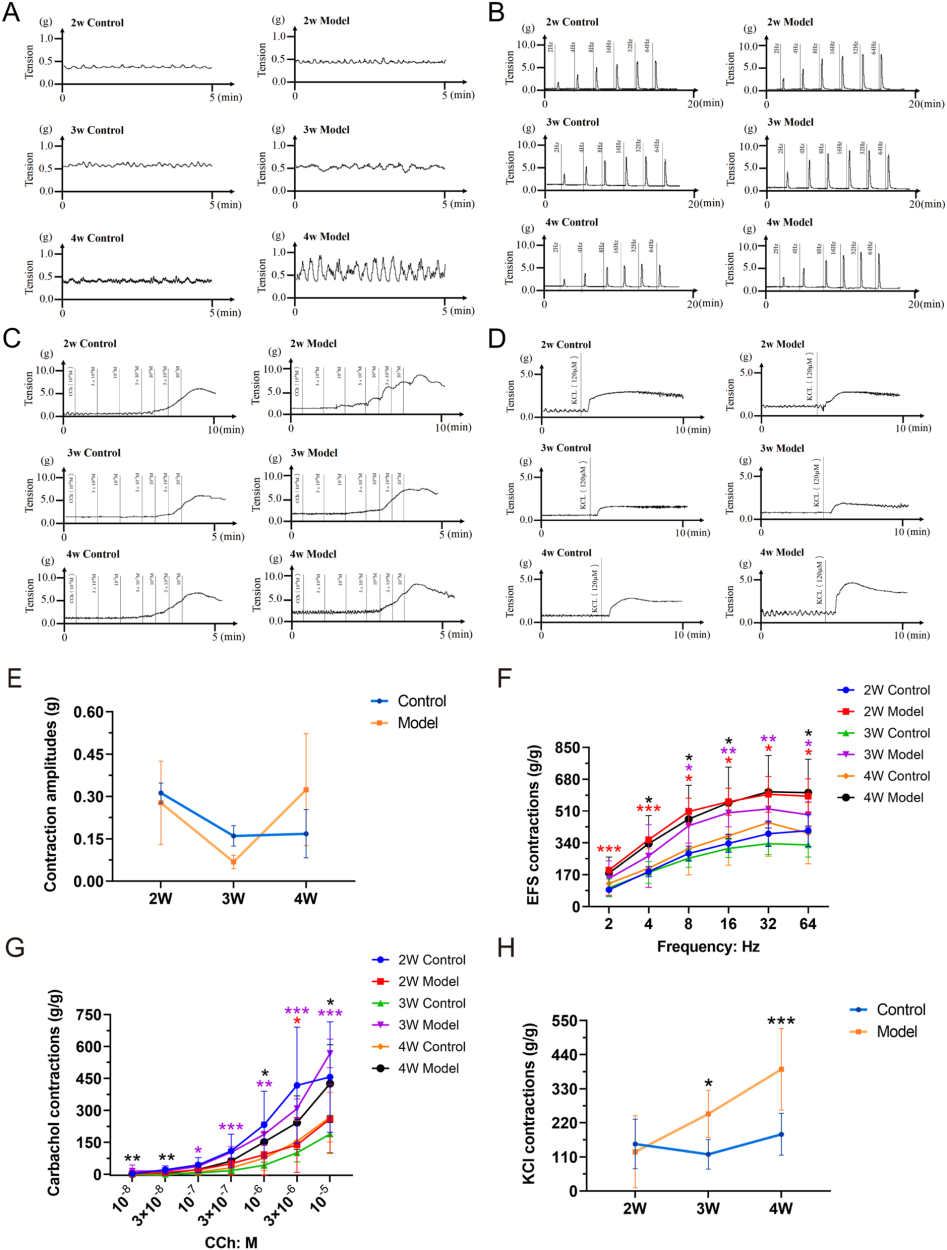
DSM contraction in response to exogenous stimulus in DCP and control rats. **A** Tension curve of spontaneous activity in detrusor smooth muscle (DSM); **B** Tension curve of response to EFS (2 – 64 Hz); **C** Tension curve of response to CCh (10^–8^ – 10^–5^ M); **D** Tension curve of response to KCl (120 mM); **E** Amplitudes of spontaneous activity in detrusor smooth muscle (DSM) (n = 4–6 rats/group for 2W, 3W, 4W, respectively); **F** DSM contraction in response to EFS (n = 3–4 rats/group for 2W, 3W, 4W respectively); **G** DSM contraction in response to CCh (n = 4–8 rats/group for 2W, 3W, 4W, respectively); **H** DSM contraction in response to KCl (n = 4–6 rats/group for 2W, 3W, 4W, respectively). Data are shown as the mean ± SD ($\overline{x }$±SD). Data were analyzed using t test or Mann–Whitney’s U test. For contraction in response to EFS, *P = 0.049535, 0.011567, 0.035934 under 8, 16 and 32 Hz EFS, ***P = 0.000368, 0.000668 under 2 and 4 Hz EFS *vs.* control group at 2W, shown as red star symbol; *P = 0.016183, 0.014669 under 8 and 64 Hz EFS, **P = 0.005369, 0.007467 under 16 and 32 Hz EFS *vs.* control group at 3W, shown as purple star symbol; *P = 0.039497, 0.048410, 0.042172 and 0.010039 *vs.* control group under 4, 8, 16 and 64 Hz EFS, respectively at 4W, shown as black star symbol. For contraction in response to CCh, *P = 0.043308 *vs.* control group at a concentration of 3 × 10^−6^M at 2W shown as red star symbol, *P = 0.011889, **P = 0.001142, ***P = 0.000651, 0.000568, 0.000477 *vs.* control group at a concentration of 10^–7^, 10^–6^, 3 × 10^–7^, 3 × 10^–6^, 10^–5^ M, respectively shown as purple star symbol; at 3W, *P = 0.033272, 0.023591, **P = 0.001229, 0.002564 *vs.* control group at a concentration of 10^–6^, 10^–5^, 10^–8^, 3 × 10^–8^ M at 4W, respectively shown as black star symbol. For contraction in response to KCl, *P = 0.027098, ***P = 0.000288 *vs.* control group at 3W and 4W, respectively

Next, the contractile properties of isolated bladder strips from diabetic rats were assessed at various time points using organ bath system. Bladder contractions were elicited using electrical field stimulation (EFS) at frequencies of 2, 4, 8, 16, 32, and 64 Hz (40 V; 0.5 ms pulse duration for 10 s), as well as with KCl (120 mM) and carbachol (CCh) (10^–8^ M to 10^–5^ M). Although bladder muscle strips from DCP rats displayed abnormal contraction patterns by the fourth week post-modeling with a increase in contraction amplitude, there is no significantly changes between the two groups (Fig. 2A and E). The contractile tension of detrusor strips in the model group was significantly elevated at the second and fourth weeks under EFS (*P* < 0.05) (Fig. 2B and F). By the third week, detrusor strips from the model group exhibited significant deviations from control under high-frequency EFS. CCh-induced contraction tension in bladder muscle strips from DCP rats was notably increased during the third and fourth weeks, particularly at higher concentrations (10^–6^ to 10^–5^ M) (*P* < 0.05) (Fig. 2C and G). Additionally, KCl stimulation revealed significant differences in detrusor strip tension in the model group by the third week, with peak tension observed in the fourth week (*P* < 0.05) (Fig. 2D and H).

### Progressive deterioration of voiding function and onset of bladder overactivity in diabetic rats by the fourth week

**Fig. 3:**
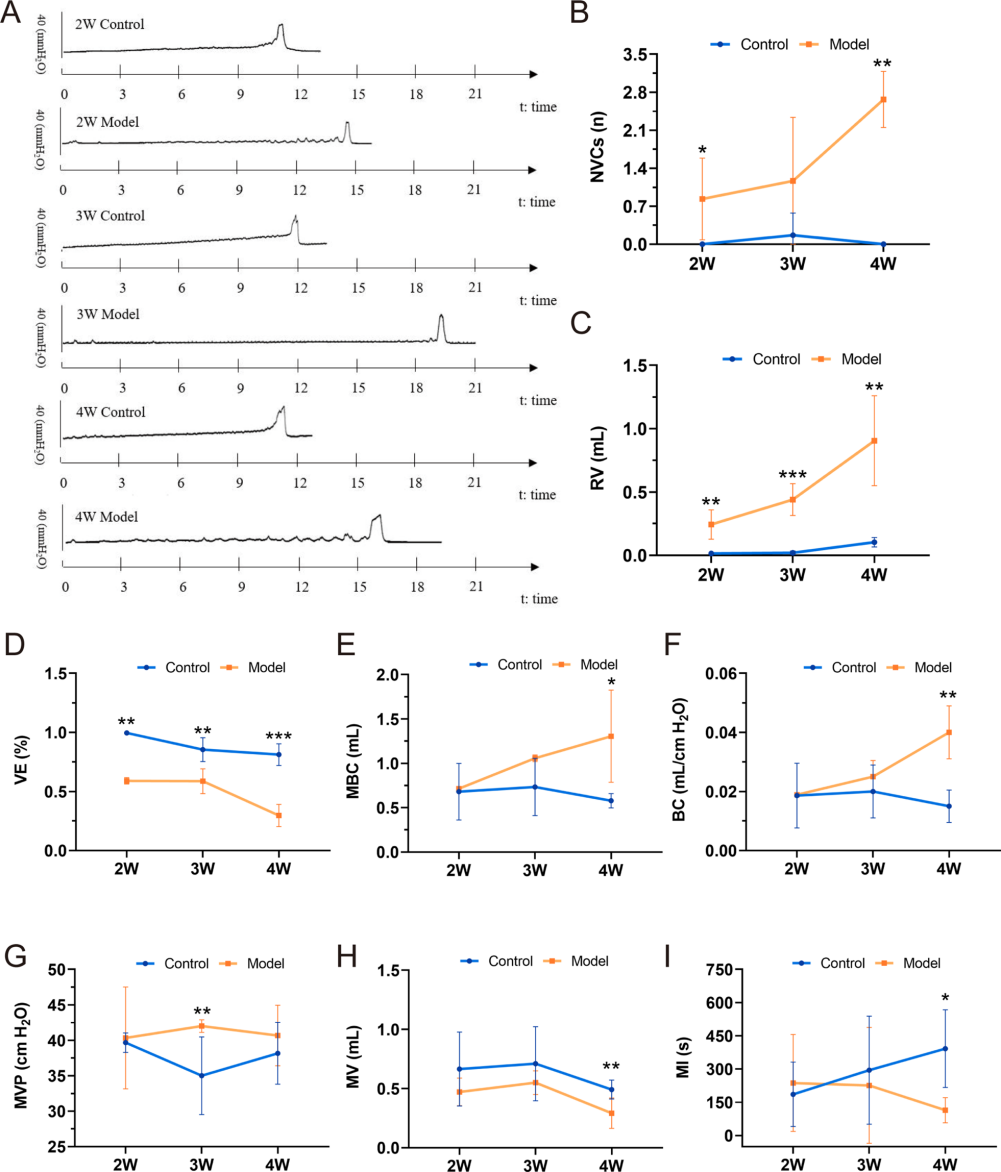
Representative cytometric recording from DCP and control rats at 2, 3, and 4 weeks. **A** Representative original recordings of urodynamic test from DCP rats and control rats; **B** the frequency of non-voiding contractions (NVCs); **C** residual volume (RV); **D** voided efficiency (VE); **E** maximum bladder capacity (MBC); **F** bladder compliance (BC); **G** maximum voiding pressure (MVP); **H** micturition volume (MV); **I** micturition interval (MI). Data are shown as the mean ± SD ($\overline{x }$±SD, n = 6 rats/group). Data were analyzed using t test or Mann–Whitney’s U test. In the case of NVCs test, *P = 0.020921 at 2W and **P = 0.001653 at 4W *vs.* control group. In the case of RV test, **P = 0.003289, 0.002526 at 2W and 4W respectively and ***P = 0.000431 at 3W *vs.* the control group. In the case of VE test, **P = 0.003289, 0.001132 at 2W and 3W, and ***P = 0.000002 at 4W respectively *vs.* control group. In the case of MBC test, *P = 0.018199 at 4W *vs.* control group. In the case of BC test, **P = 0.003289 at 4W *vs.* control group. In the case of MVP test, **P = 0.003289 at 3W *vs.* control group. For MV and MI, **P = 0.008839 and *P = 0.044665 at 4W *vs.* control group, respectively

Urodynamic assessment of DCP rats revealed prolonged voiding times and increased non-voiding contractions (NVCs) prior to urination compared to controls (Fig. 3A, B). The model group exhibited significantly greater residual urine volume (RV), maximum bladder capacity (MBC), compliance (BC), and maximum voiding pressure (MVP), alongside increased NVCs, while voiding efficiency (VE), micturition volume (MV), and micturition interval (MI) was markedly reduced (*P* < 0.05). Specifically, RV was significantly elevated at 2, 3, and 4 weeks post-modeling (*P* < 0.01) and progressively increased over time (Fig. 3C). VE decreased significantly at all time points, with a sharp decline by the fourth week (*P* < 0.01) (Fig. 3D), besides, the intergroup comparisons showed control animals exhibited a marginal yet statistically significant decline in VE at the fourth week compared to Week 2 (*P* < 0.05), while the model group demonstrated pronounced reductions in VE at the fourth week relative to both Week 2 and Week 3 (*P* < 0.01), with this late-stage impairment being substantially more severe than concurrent control measurements (see Additional file 1). MBC and BC also increased significantly over time in the model group compared to controls (*P* < 0.05) (Fig. 3E, F). In DCP rats, the MVP was significantly higher than that in the control group in the third week (*P* < 0.01), indicating significant hyperactivity of the DCP bladder (Fig. 3G). MV and MI gradually decreased in DCP rats, with significant differences emerging in the fourth week (*P* < 0.05) (Fig. 3H, I). The urination intervals in DCP rats gradually decreased, and the urine volume per void also decreased. These findings demonstrate that diabetic cystopathy in the rats progressively exacerbated over time, ultimately resulting in the onset of bladder overactivity by the fourth week.

### Identification of Myosin 5a as a key mediator in the pathophysiological mechanisms of OAB through public database mining

**Fig. 4:**
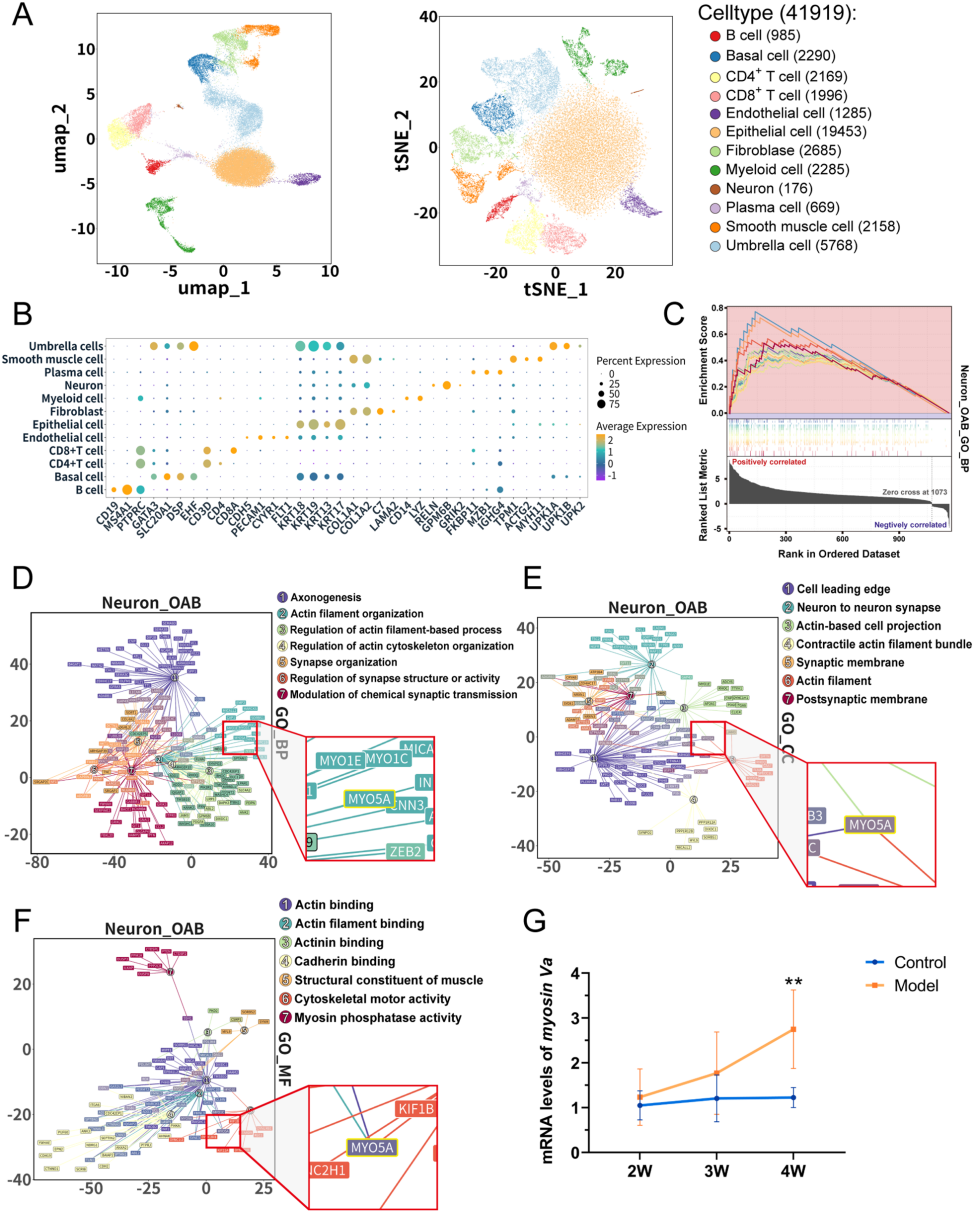
Analysis of scRNA-seq data from patients with clinical bladder overactivity. **A** UMAP and t-SNE plots of cells from the 7 human bladder samples profiled in this study. UMAP (Uniform Manifold Approximation and Projection) and tSNE (t-Distributed Stochastic Neighbor Embedding) are both dimensionality reduction techniques used to visualize scRNA-seq data; **B** Canonical marker genes for bladder cell subsets; **C** Line chart showing the results of GSEA enrichment pathways for Neuron in the OAB group (NES > 1.50, *P*.adjust < 0.05).; **D** Network plot showing enrichment GO terms for Neuron in OAB group (Biological Process); **E** Network plot showing enrichment GO terms for Neuron in OAB group (Cell Component); **F** Network plot showing enrichment GO terms for Neuron in OAB group (Molecular Function); **G** Changes in mRNA expressions of Myosin 5a at 2, 3, and 4 weeks in rats’ bladder tissue. Data are shown as the mean ± SD ($\overline{x }$±SD, n = 6 rats/group). Data were analyzed using t test or Mann–Whitney’s U test. **P = 0.002035 at 4W vs. control group

To explore the factors contributing to bladder dysfunction under OAB versus normal conditions, we incorporated clinical single-cell RNA-sequencing data from three distinct groups of bladder samples for analysis. A total of 41,919 cells were included for subsequent analysis, during which 11 major cell types were identified, including B cell, basal cell, endothelial cell, epithelial cell, fibroblast, myeloid cell, neuron, plasma cell, smooth muscle cell, T cell, and umbrella cell (Fig. 4A, B). Preliminary enrichment analysis of differentially expressed genes (DEGs) between the OAB and normal bladder revealed the activation of actin-regulating pathways in bladder neurons under OAB conditions, encompassing the integration and genesis of synaptic structures (see Additional file 1, 2). Subsequently, Gene Set Enrichment Analysis (GSEA) specifically on DEGs of OAB bladder neurons indicated a propensity for the activation of biological activities associated with neuronal growth and axonal formation in the bladder under dysfunctional conditions (Fig. 4C, see Additional file 1, 3). Building on these findings, a detailed Gene Ontology (GO) enrichment analysis revealed significant tendencies for actin-related pathway enrichment across all three aspects (Biological Process, Cellular Component, and Molecular Function), such as Actin filament organization, Cell leading edge, Actin-based cell protection, and Cytoskeletal motor activity (Fig. 4D–F). Notably, Myosin 5a (MYO5A in Homo sapiens) was found to be involved in all these pathways, suggesting that in patients with OAB, bladder neurons exhibit abnormally heightened activity in the integration of actin and regulation of synaptic signal transmission, implying that Myosin 5a may be one of the key factors in the dysregulation of bladder neuron modulation.

### Impact of Myosin 5a on neurotransmitter-associated protein ChAT, SP, and VIP levels in rats with diabetic cystopathy

**Fig. 5:**
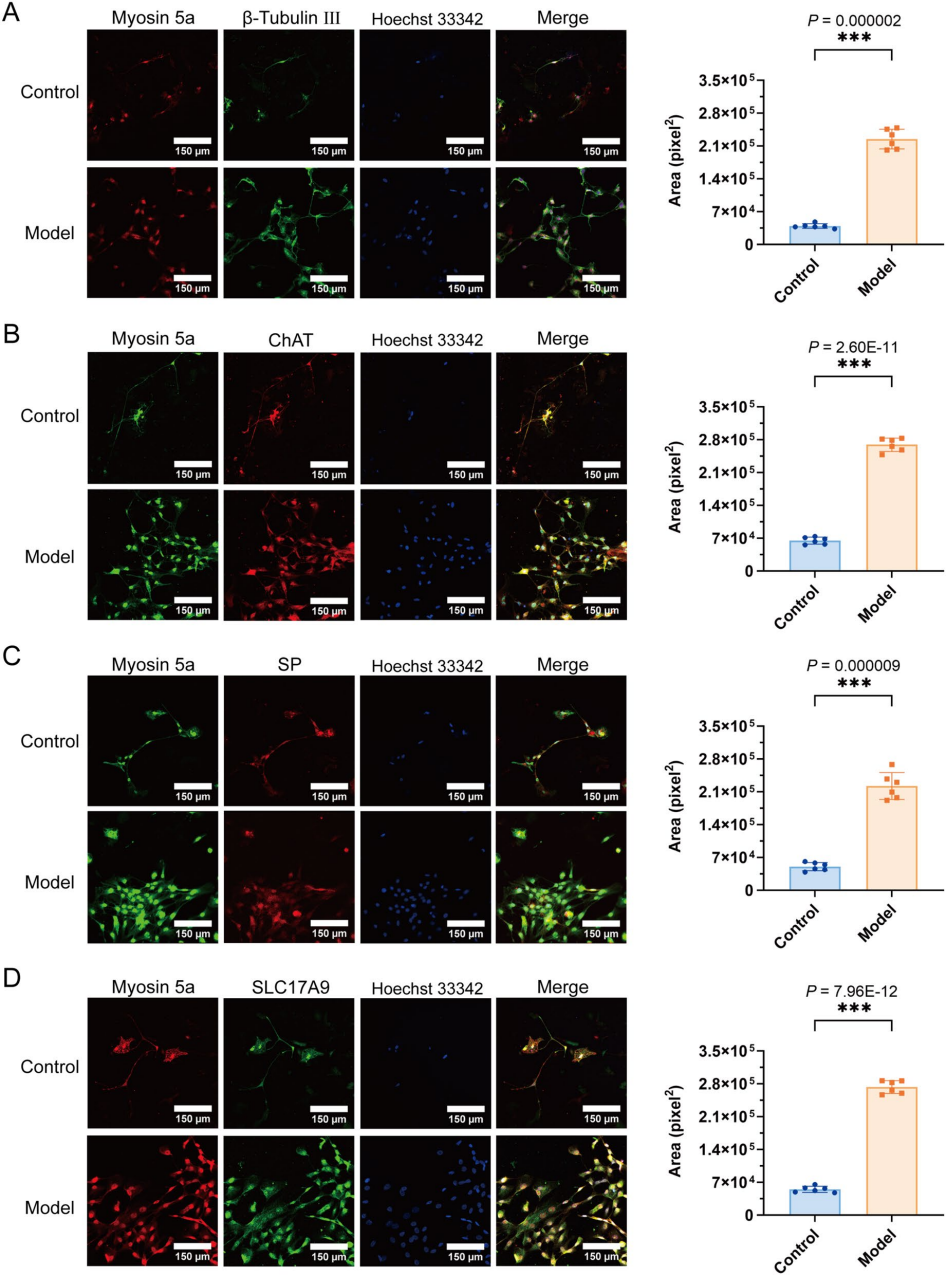
Cell immunofluorescence staining in DCP and control rats. **A** Co-localization of Myosin 5a with β-tubulin and expression area statistics of β-tubulin in DCP bladder neurons; **B** Co-localization of Myosin 5a with ChAT and expression area statistics of ChAT in DCP bladder neurons; **C** Co-localization of Myosin 5a with SP and expression area statistics of SP in DCP bladder neurons; **D** Co-localization of Myosin 5a with SLC17A9 and expression area statistics of SLC17A9 in DCP bladder neurons. Data are shown as the mean ± SD ($\overline{x }$±SD, n = 6 rats/group). Data were analyzed using t test. ***P = 0.000002, 2.60E-11, 0.000009, 7.96E-12 *vs.* control group for expression area statistics of β-tubulin, ChAT, SP, SLC17A9, respectively

**Fig. 6:**
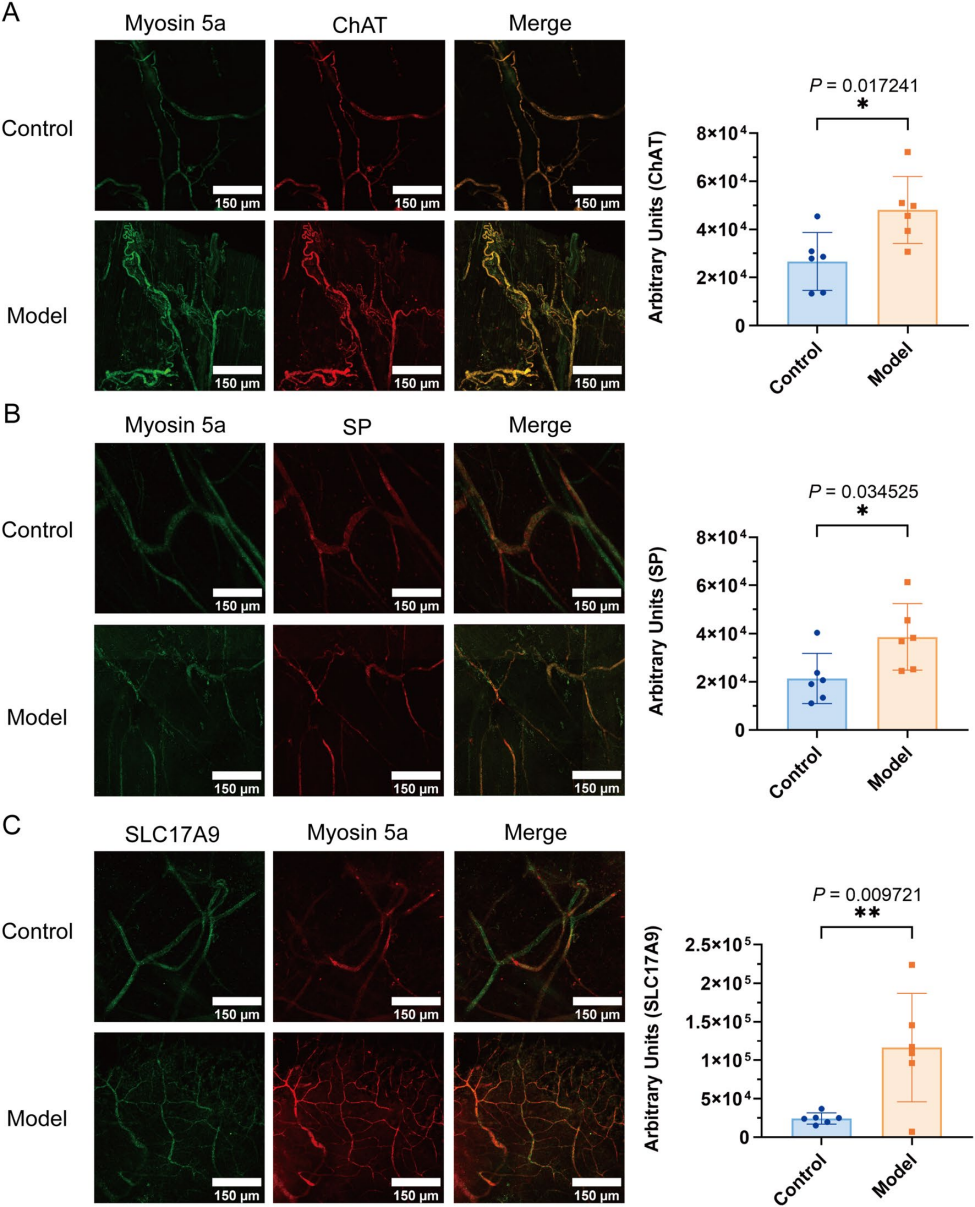
Tissue immunofluorescence staining in DCP and control rats at 4 weeks after administration. **A** Co-localization of Myosin 5a with ChAT and expression area statistics of ChAT in DCP bladder tissues; **B** Co-localization of Myosin 5a with SP and expression area statistics of SP in DCP bladder neurons; **C** Co-localization of Myosin 5a with SLC17A9 and expression area statistics of SLC17A9 in DCP bladder tissue. Data are shown as the mean ± SD ($\overline{x }$±SD, n = 6 rats/group). Data were analyzed using t test. *P = 0.017241, 0.034525, **P = 0.009721 *vs.* control group for expression area statistics of ChAT, SP, SLC17A9, respectively

Based on the aforementioned data, we hypothesize that the progression of diabetic cystopathy is associated with the levels of Myosin 5a and bladder neurotransmitters. Therefore, we conducted RT-qPCR to assess the expression of Myosin 5a in the bladder at different time points. We observed a progressive increase in Myosin 5a expression over time, with a peak at the fourth week, which correlates positively with the advancement of diabetic bladder dysfunction (Fig. 4G). Analysis of primary bladder neurons revealed that Myosin 5a colocalizes with neurotransmitter-associated proteins βIII-tubulin, ChAT, SP, and VIP. Furthermore, Myosin 5a was shown to upregulate the expression of these proteins (*P* < 0.05). These findings were corroborated by immunofluorescence assay of whole bladder tissue sections. (Figs. 5 and 6).

### Myosin 5a siRNA knockdown decreased expression of ChAT and SP in bladder neurons

**Fig. 7:**
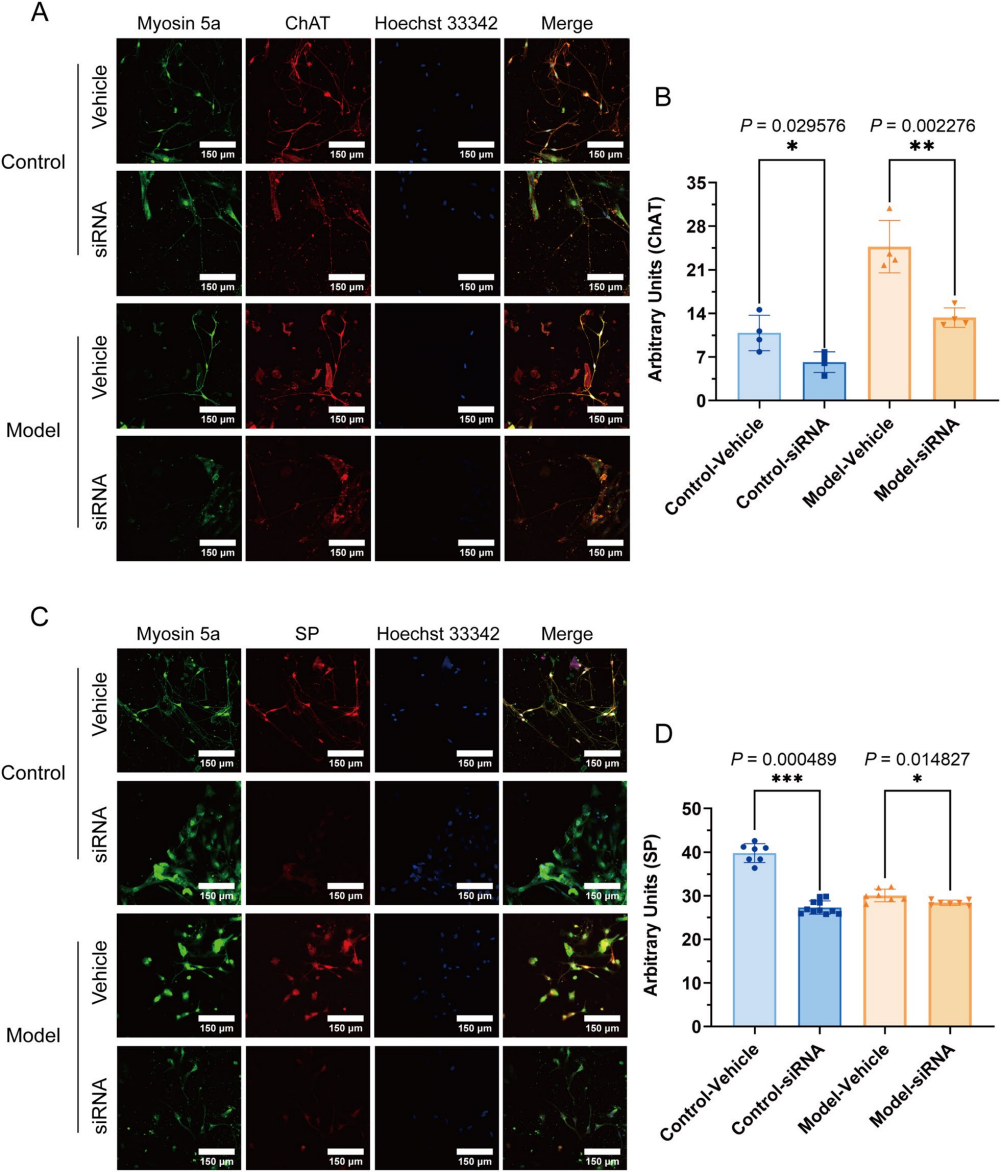
Changes of ChAT, SP expression in bladder neurons after interference with Myosin 5a at 4 weeks after administration. **A** Immunofluorescence analysis of bladder neurons stained with anti-ChAT after interference with Myosin 5a; **B** Quantitative summary of the distribution of ChAT in bladder neurons after interference with Myosin 5a; **C** Immunofluorescence analysis of bladder neurons stained with anti-SP after interference with Myosin 5a; **D** Quantitative summary of the distribution of SP in bladder neurons after interference with Myosin 5a. Data are shown as the mean ± SD ($\overline{x }$±SD, n = 4 rats/group for Immunofluorescence analysis stained with anti-ChAT; for Immunofluorescence analysis stained with anti-SP, n = 11 rats in control-siRNA group, n = 7 rats in other groups). Data were analyzed using t test or Mann–Whitney’s U test. For analysis stained with anti-ChAT, comparison within control group, *P = 0.029576 *vs.* vehicle group; comparison within model group, **P = 0.002276 *vs.* vehicle group. For analysis stained with anti-SP, comparison within control group, ***P = 0.000489 *vs.* vehicle group; comparison within model group, *P = 0.014827 *vs.* vehicle group

To further investigate the role of Myosin 5a in bladder excitatory neurotransmitters, we performed knockdown experiments targeting Myosin 5a expression in various neuronal populations. The results demonstrated that the knockdown of Myosin 5a significantly reduced the expression of the cholinergic neurotransmitter-related protein ChAT, with a notably greater reduction in the model group compared to the control group. Additionally, the expression of the excitatory neurotransmitter SP was significantly diminished, though the reduction in the model group was less pronounced relative to the control group. These findings suggest that, in diabetic cystopathy, Myosin 5a exerts a more substantial influence on cholinergic neurotransmission than on peptidergic pathways (*P* < 0.05) (Fig. 7).

### Urinary dysfunction in Myosin 5a-deficient mice

**Fig. 8:**
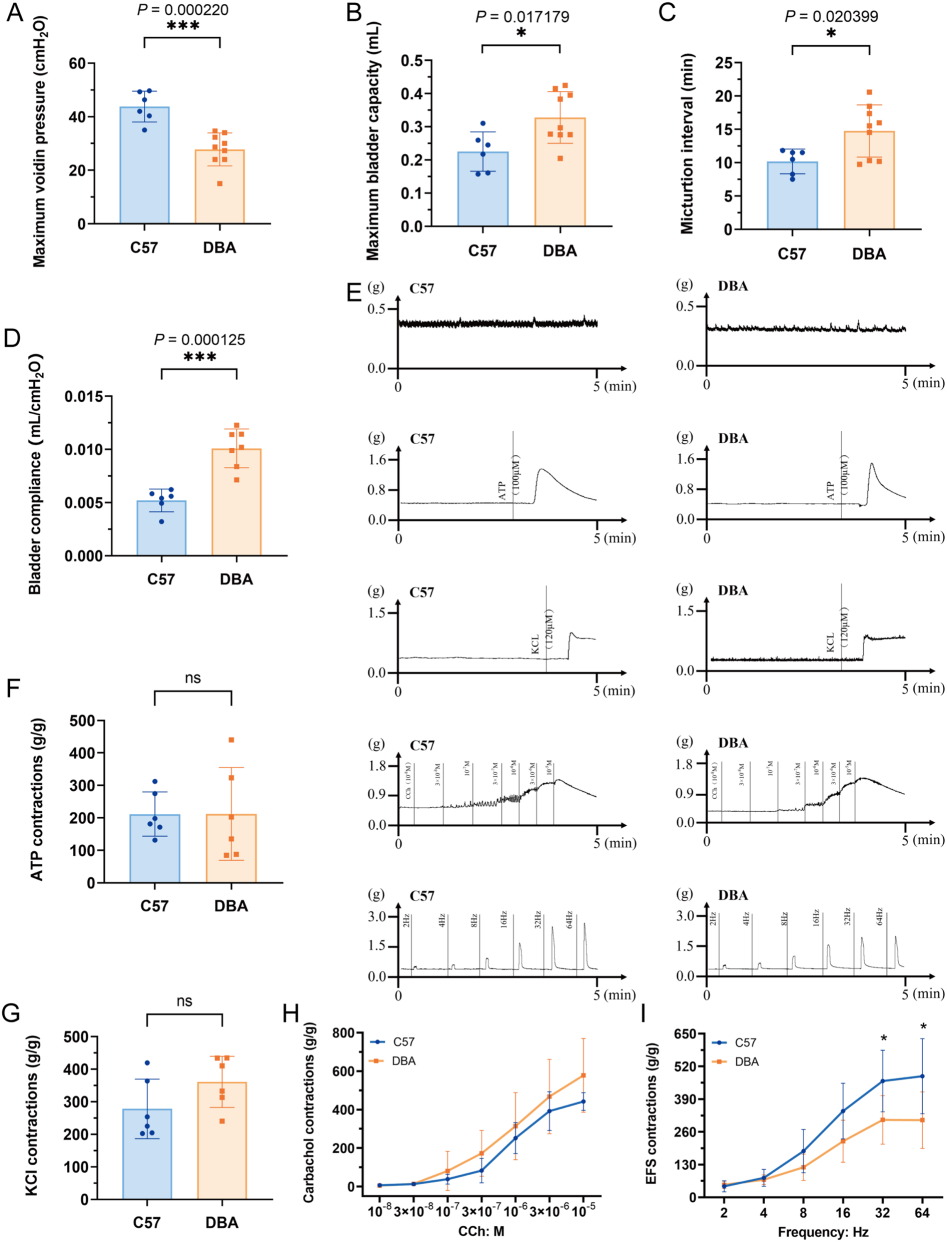
DSM contraction in response to exogenous stimulus in DBA and WT mice. **A** Maximum voiding pressure (MVP); **B** Maximum bladder capacity (MBC); **C** Micturition interval; **D** bladder compliance (BC); **E** Tension curve in detrusor smooth muscle (DSM) of DBA and WT mice (from top to bottom: spontaneous activity, response to α, β-MeATP, KCl (120 mM), CCh (10^–8^ -10^–5^ M), EFS (2–64 Hz)); **F** DSM contraction in response to ATP of in DBA and WT mice; **G** DSM contraction in response to KCl (120 mM) in DBA and WT mice;** H** DSM contraction in response to CCh (10^–8^ -10^–5^ M) in DBA and WT mice;** I** DSM contraction in response to EFS (2–64 Hz) in DBA and WT mice. Data are shown as the mean ± SD ($\overline{x }$±SD, n = 6 mice/WT group, n = 9 mice/DBA group in MVP and micturition interval test, n = 7 mice in DBA group in BC test and n = 6 mice in DBA group in the Tension test). Data were analyzed using t test or Mann–Whitney’s U test. In the case of MVP test, ***P = 0.000220 *vs.* WT group. In the case of MBC test, *P = 0.017179 *vs.* WT group. In the case of micturition interval test, *P = 0.020399 *vs.* WT group. In the case of BC test, ***P = 0.000125 *vs.* WT group. For contraction in response to ATP, KCl and CCh, there’s no significant change between two groups. For contraction in response to EFS, *P = 0.035631, 0.045726 *vs.* WT group under 32 Hz and 64 Hz, respectively

Mice with complete Myosin 5a deficiency, due to *Myosin 5a* gene deletion (dilute-lethal mice), die in infancy. In contrast, DBA mice (dilute, brown, non-agouti), which have a partial Myosin 5a deficiency caused by a viral insertion in the *Myosin 5a* gene, survive into adulthood. Therefore, we conducted urodynamic assessments in DBA mice to investigate the impact of Myosin 5a deficiency on urinary function. Urodynamic test results show that the maximum voiding pressure of the model group mice significantly decreased (*P* < 0.001) (Fig. 8A), whereas the maximum bladder capacity, micturition intervals and bladder compliance, markedly increased, compared with those of the control group mice (*P* < 0.05) (Fig. 8B–D).

### Decreased response of isolated bladder strips to EFS, CCh, and KCl stimulation in Myosin 5a-deficient mice

Bladder strips were allowed to balance for 45 min before inducing contractions using α, β-methylene ATP (100 μM), EFS (2–64 Hz), KCl (120 mM), and carbachol (10^−8^ M to 10^−5^ M). The detrusor strips in DBA mice showed a lower amplitude compared to those in C57 mice (Fig. 8E). However, there were no significant differences between the two groups in response to α, β-methylene ATP, KCl, and CCh. Notably, contractions of the bladder strips in response to high-frequency EFS (32 Hz, 64 Hz) were significantly lower in the model group than in the control group (*P* < 0.05) (Fig. 8I).

### Alterations in neurotransmitter-associated protein levels in the bladder of Myosin 5a-deficient mice

**Fig. 9:**
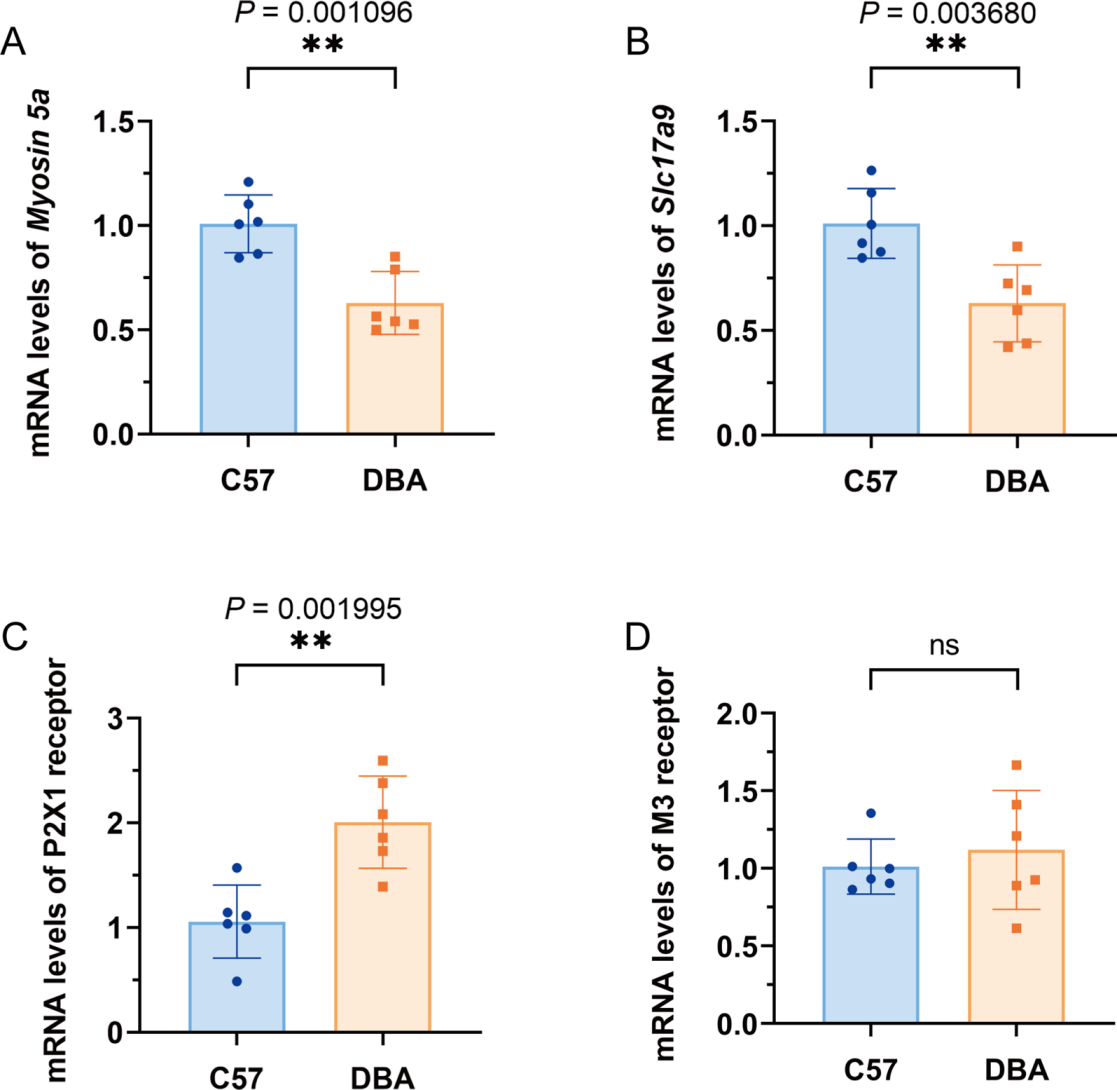
Urodynamic parameters and relative gene expression in bladder from DBA and WT mice. **A** *Myosin 5a* gene-expression levels measured by qPCR in bladder; **B** *Slc17a9* gene-expression levels measured by qPCR in bladder; **C** P2X1 receptor gene-expression levels measured by qPCR in bladder; **D** M3 receptor gene-expression levels measured by qPCR in bladder. Data are shown as the mean ± SD ($\overline{x }$±SD, n = 6 mice/group in the qPCR test). Data were analyzed using t test or Mann–Whitney’s U test. **P = 0.001096, 0.003680, 0.001995 *vs.* WT group in *Myosin 5a, Slc17a9* and P2X1 receptor mRNA, while there’s no significant change in expression of M3 receptor

Real-time PCR analysis reveals significant down-regulation of *Myosin 5a* and *Slc17a9* gene expression in the model group compared to the control group (*P* < 0.001) (Fig. 9A, B). On the other hand, the expression levels of the P2X1 receptor in DBA mice were significantly higher than in C57 mice (*P* < 0.01) (Fig. 9C). However, there were no significant differences in the gene expression levels of M3 receptors between the two groups (*P* > 0.05).

## Discussion

Hyperglycemia damages neurons through multiple pathways, including sorbitol and fructose accumulation, oxidative stress, protein kinase C activation, and advanced glycation end products (Sies et al. 2005). Using an STZ-induced diabetic rat model, we observed increased bladder sensitivity to stimuli and confirmed bladder overactivity by the fourth week. EFS stimulation mimics in vivo bladder nerve activation. DCP rats showed stronger detrusor contractions, suggesting impaired nerve conduction and overactive bladder. The parasympathetic system regulates bladder contraction via M3 and M2 receptors, counteracting β-adrenoceptor-induced relaxation (Yamazaki et al. 1998; Michel and Sand 2009; Plantone and Koudriavtseva 2016). Carbachol stimulation further enhanced detrusor responses in DCP rats, indicating abnormal cholinergic signaling. Voltage-gated potassium channels (VGKCs) regulate bladder contractions. High KCl concentrations trigger neurotransmitter release, including nitric oxide and acetylcholine (Chataigneau et al. 1999; Munoz et al. 2011). DCP rat bladders showed heightened KCl-induced contractions, consistent with clinical findings. Early-stage diabetic rats also exhibit increased acetylcholine release, higher muscarinic receptor density, and elevated urothelial ATP release, suggesting detrusor hyperactivity may initially compensate for increased urine production in diabetes (Munoz et al. 2011; Meng et al. 2012).

Myosin motors are part of a large superfamily of motor proteins with unique molecular structures designed to transport specific cargoes, facilitating a wide range of physiological activities. Myosin 5a is an extensively distributed motor found in both presynaptic and postsynaptic neurons (Bridgman 2004; Kneussel and Wagner 2013). Under normal physiological conditions, bladder contraction primarily involves purinergic and cholinergic pathways. The release of neurotransmitters is a complex process that requires specific secretory vesicles to store and transport the neurotransmitters before their release into the synaptic cleft (Chaudhry et al. 2008). In recent studies, Myosin 5a has been found to play a crucial role in the transport of purinergic and cholinergic neurotransmitters (Chaudhury et al. 2012; Takagishi et al. 2005b). Additionally, SLC17A9 has been identified as a vesicular nucleotide transporter that plays a vital role in transporting the purinergic neurotransmitter ATP to specific secretory vesicles (Hasuzawa et al. 2020). Furthermore, Ira V. Röder's research demonstrated that Myosin 5a cooperates with PKA on the postsynapse to regulate the size and integrity of synapses, as well as the lifetime of neurotransmitter receptors (Röder et al. 2010). In present study, we found that after diabetes modeling, the expression of Myosin 5a in the bladder gradually increased over time, reaching a peak at the fourth week, and the animals had OAB symptoms. Strikingly, immunofluorescence results showed that Myosin 5a appears co-localized with excitatory neurotransmitters-related proteins such as ChAT, SLC17A9, SP, indicating that Myosin 5a may further affect nerve conduction due to such a spatial relationship. After further statistics, the number of ChAT-, SLC17A9-, and SP- positive neurons increased with Myosin 5a. We hypothesized that Myosin 5a may affect bladder nerve conduction, and OAB may also be caused by the surge of Myosin 5a expression. To shed light on the regulation of Myosin 5a to the nerve conduction, we employed interfering with Myosin 5a to find that ChAT-, SLC17A9-, and SP- positive neurons decreased as we expected, this strongly suggests the existence of a functional link between the density of Myosin 5a and bladder nerve conduction.

Mutations in the *Myosin 5a* gene cause the human neurological disorder, type 1 Gricecelli syndrome. In addition to distinctive skin and hair color, patients with type 1 Gricecelli syndrome involve severe primary neurological deficits, and affected individuals often exhibit hypotonia, a phenomenon seen in the bladders of DBA mice (Carew et al. 2022). Carew's research focused more on the relationship between myosin5a and cholinergic neurotransmitters in the bladder of DBA mice with myosin5a deficiency. However, it did not delve into the urination function of DBA mice or the correlation between myosin5a and peptidergic or purinergic nerves. This study precisely fills these gaps (Carew et al. 2022). In the urodynamic evaluation, the voiding pressure of DBA mice was significantly reduced, and the bladder showed high compliance, and the longer voiding interval was also echoed with the results of increased bladder capacity, which may be due to the partial deletion of Myosin 5a. Sensory neuron dysfunction, resulting from insensitivity to stretch-induced bladder volume expansion, has not been explored in depth here (Griscelli et al. 1978).

The bladder's normal physiological function is primarily regulated by the cholinergic and purinergic pathways. Muscle tension experiments using exogenous receptor agonists and electrical field stimulation (EFS) help assess bladder muscle strip contraction and relaxation. EFS mimics neurogenic contraction by triggering neurotransmitter release from motor neurons to effector organs, inducing smooth muscle contractions.

DBA/2J mice showed weaker detrusor contractions than C57BL/6 mice under high-frequency EFS, despite similar responses to exogenous agonists. This implies impaired neurotransmitter release or endogenous receptor dysfunction in DBA/2J mice, consistent with their reduced EFS-induced smooth muscle tension in gastric and corpus cavernosum tissues (Chaudhury et al. 2014). Voltage-gated potassium channels activity depends on membrane potential. High extracellular potassium lowers resting potential, enhancing excitability. Normal KCl-induced contractions in DBA/2J mice suggest intact VGKC function despite partial Myosin 5a loss. Intriguingly, P2X1 receptor expression was upregulated, potentially compensating for reduced neurotransmitter release during EFS.

Diabetic rats’ bladder overactivity may involves two mechanisms: denervation supersensitivity and increased muscarinic receptors, with Myosin 5a connecting both processes. Although early hyperactivity stems from denervation supersensitivity (nerve loss and heightened neurotransmitter sensitivity), Myosin 5a worsens this by controlling vesicle transport. Single-cell data show Myosin 5a works with SLC17A9, and mice lacking Myosin 5a had lower SLC17A9 levels and weaker sensory responses. This suggests diabetes-induced Myosin 5a increases neurotransmitter release (ATP/acetylcholine), accelerating bladder sensitivity before nerve damage becomes severe.

This presynaptic hyperactivity may combine with known M2/M3 receptor increases (Tong et al. 2002; Tong and Cheng 2002). Reducing Myosin 5a lowered ChAT (makes acetylcholine) and SP (pain-signaling neurotransmitter) in bladder nerves, indicating it maintains neurotransmitter levels. Diabetes-induced Myosin 5a overexpression likely causes neurotransmitter buildup, forming a cycle that boosts postsynaptic receptors. This explains diabetic bladder disease progression: early nerve overactivity via Myosin 5a prepares the bladder muscle for later receptor-driven overaction as nerves deteriorate. By connecting Myosin 5a to both nerve signaling and receptor changes, we identify it as a key target to break the damaging nerve-muscle cycle in diabetic bladder disease.

In the present study, there were some limitations that need to be considered. First, we did not assess the pathways through which Myosin 5a affects neurotransmitter expression. The possible explanation is that it affects the transport of synaptic vesicles and the circulation of postsynaptic membrane receptors. Secondly, it is important to note that animal models and underlying mechanisms of different DCP models may vary. While the STZ model provides some resemblance to DCP in patients, it cannot fully replicate all the physiological and pathological conditions observed in human patients with DCP. Despite this limitation, our study provides strong evidence supporting the role of Myosin 5a in bladder regulation in DCP rats. Further research and investigations are warranted to delve deeper into this area and explore additional aspects of DCP pathophysiology.

## Conclusions

In conclusion, through a series of in vitro and in vivo studies, we demonstrated that Myosin 5a plays a crucial function in the regulation of bladder motor nerves, and abnormal expression of Myosin 5a in STZ-induced diabetic rats may affect bladder neurotransmission and aggravate overactivity bladder. The scientific understanding also provides a pathophysiological basis for the study of clinical treatment of OAB caused by diabetes. At the same time, it provides more complete research methods and ideas for new drugs targeting Myosin 5a.

## Supplementary Information

Supplementary Material 1.

Supplementary Material 2.

Supplementary Material 3.

## Data Availability

The raw data supporting the conclusions of this manuscript will be made available by the authors, without undue reservation, to any qualified researcher. The public datasets (GSM4850577, GSM5733428, and GSE175526 (dataset GSM5340862-GSM5340866)) can be downloaded from the NCBI-GEO database (http://www.ncbi.nlm.nih.gov/geo/). The code relevant to this article can be found on the official SCP/Seurat website (https://zhanghao-njmu.github.io/SCP/index.html#dynamic-features, https://satijalab.org/seurat/).

## Abbreviations

Ach: Acetylcholine
ATP: Adenosine triphosphate
BC: Bladder compliance
CCH: Carbachol
ChAT: Choline acetyltransferase
DCP: Diabetic cystopathy
DM: Diabetes mellitus
EFS: Electrical-field stimulation
FBG: Fasting blood glucose
GO: Gene Ontology
KEGG: Kyoto Encyclopedia of Genes and Genomes
MBC: Maximum bladder capacity
MVP: Maximum voiding pressure
NVCs: Frequency of non-voiding contractions
OAB: Overactive bladder
PBS: Phosphate-buffered saline
RT: Room temperature
RV: Residual volume
scRNA-seq: Single-cell RNA sequencing
SD: Sprague–Dawley
SP: Substance P
STZ: Streptozotocin
SLC17A9: Solute carrier family 17 member 9
VE: Voided efficiency
