# Supplementary material for: Imbalance of bladder neurohomeostasis by Myosin 5a aggravates diabetic cystopathy
Source: Mol Med. 2025 Mar 10;31:91. doi: 10.1186/s10020-025-01140-6 (PMC11892272; doi:10.1186/s10020-025-01140-6)
Supplement: Supplementary file 1 — Supplementary Material 1. [file 10020_2025_1140_MOESM1_ESM.docx]

Additional file 1


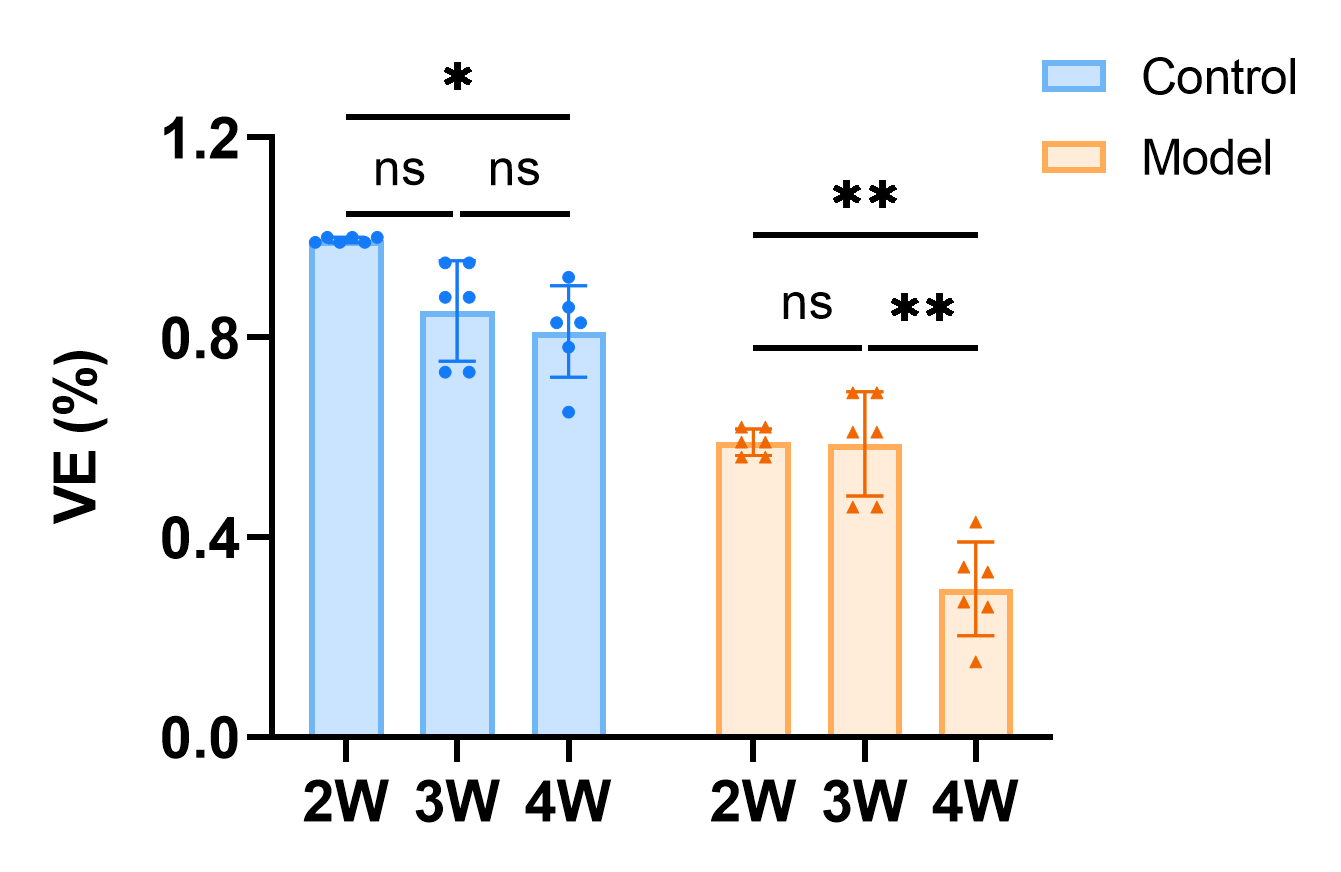


Additional file 1. Intergroup comparative analysis of voiding efficiency (VE) across three timepoints revealed distinct temporal patterns.

n=6 rats/group. Data are shown as the mean ± SD ($\bar{x}$±SD). Data were analyzed using t test or Mann-Whitney’s U test. For control group, **P* = 0.013134 in the comparison between 4W and 2W; for model group, ***P* = 0.001141, 0.001543 in the comparison between 4W and 2W or 3W, respectively.


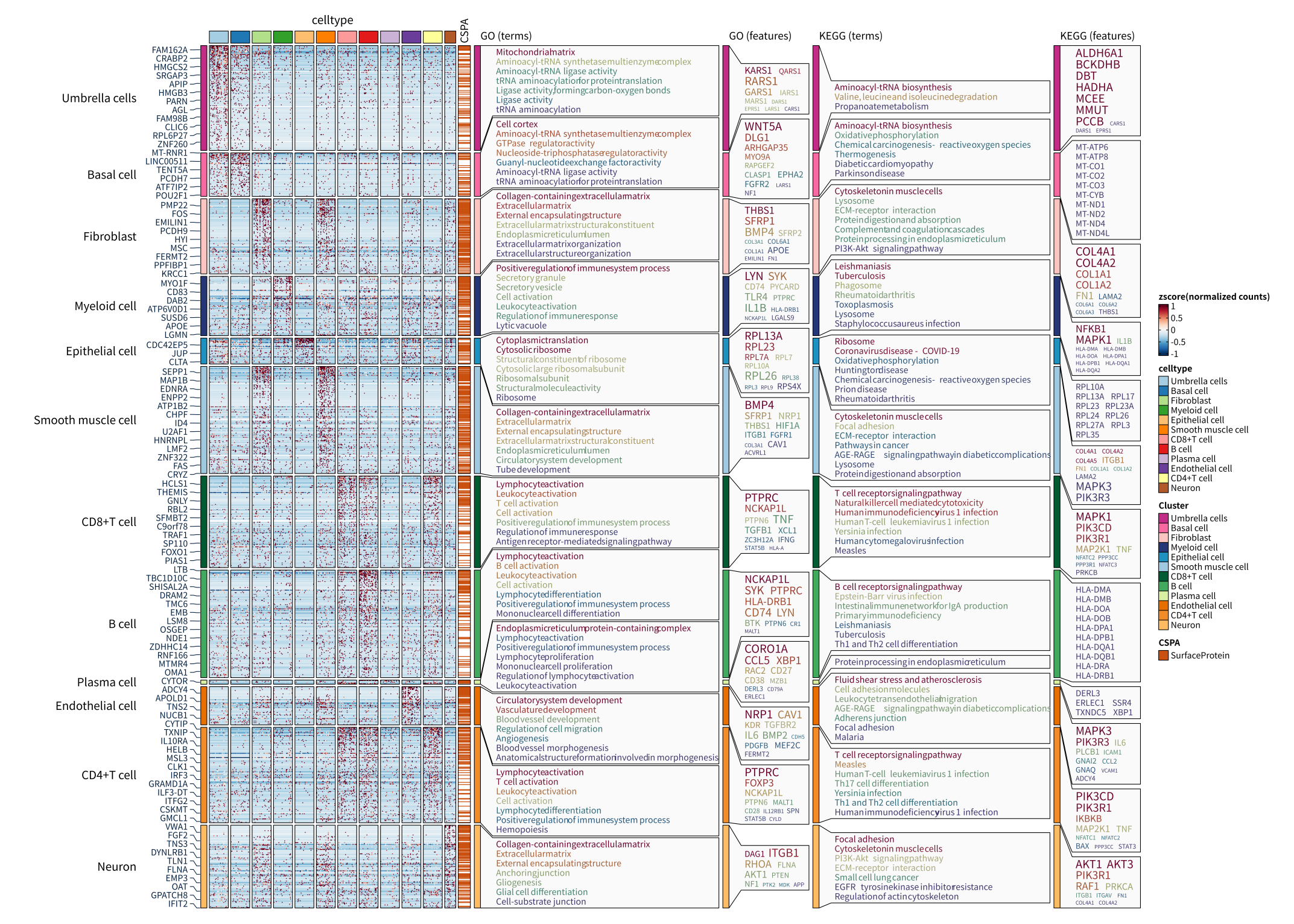


Additional file 2. Results of the scRNA-seq GO/KEGG/CSPA analysis from clinical bladder samples.


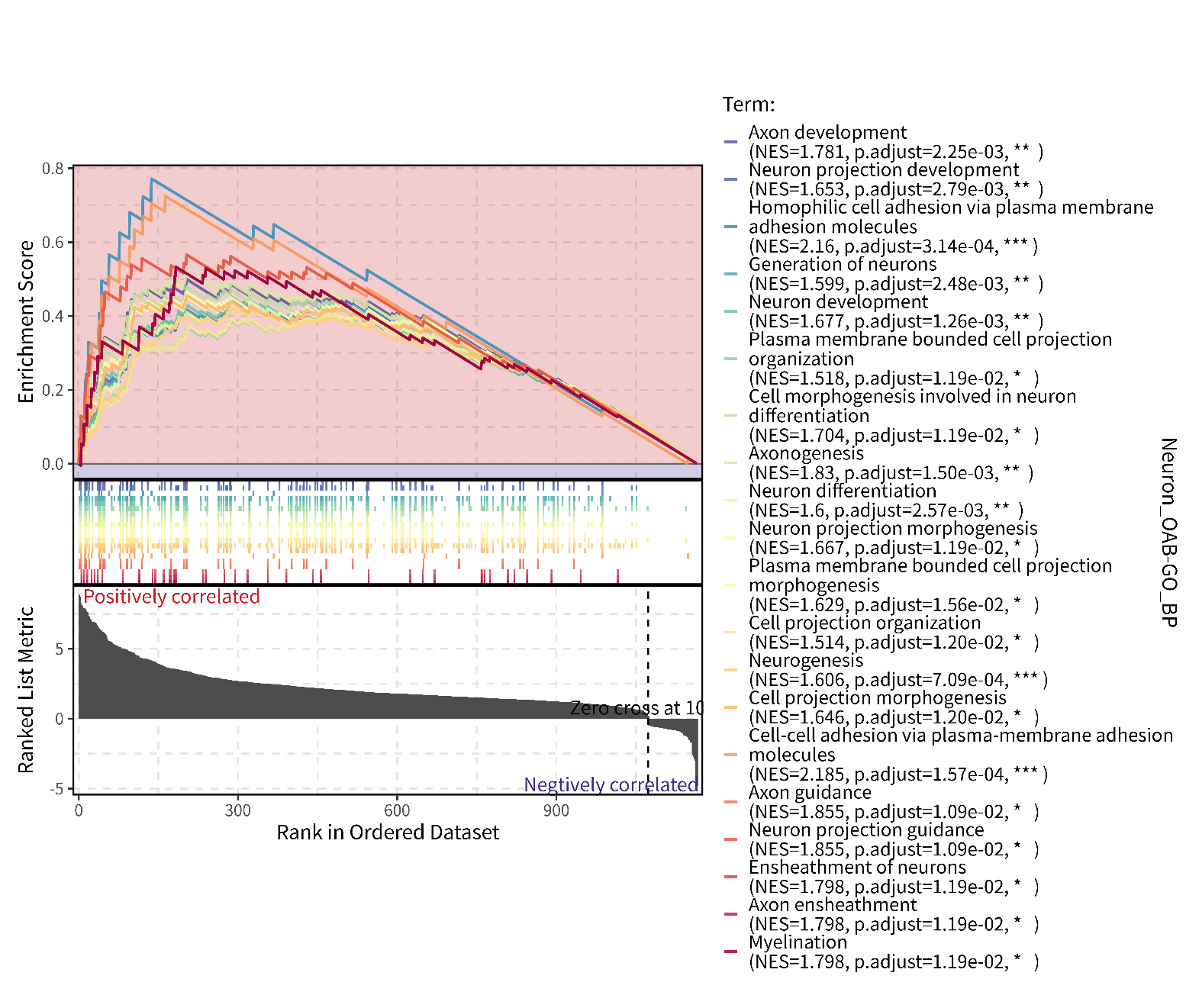


Additional file 4. Result of the Gene Set Enrichment Analysis for Neuron in OAB group from clinical bladder samples.
